# Supplementary material for: A hierarchical modelling approach to assess multi pollutant effects in time-series studies
Source: PLoS One. 2019 Mar 4;14(3):e0212565. doi: 10.1371/journal.pone.0212565 (PMC6398830; doi:10.1371/journal.pone.0212565)
Supplement: S1 File — It includes a series of figures and tables related to descriptive statistics, convergence of the MCMC simulation and sensitivity analysis results. (PDF) [file pone.0212565.s001.pdf]

Descriptives

The plots in Figures A–C present the relationship between the meteorological variables (temperature and relative humidity) and the measured concentrations of the six pollutant metrics. Due to the presence of a degree of non linearity in the exposure model, we have included the quadratic term for the two meteorological variables.

The plots in Figures D–I present the the time-series of mortality and of each pollutant.

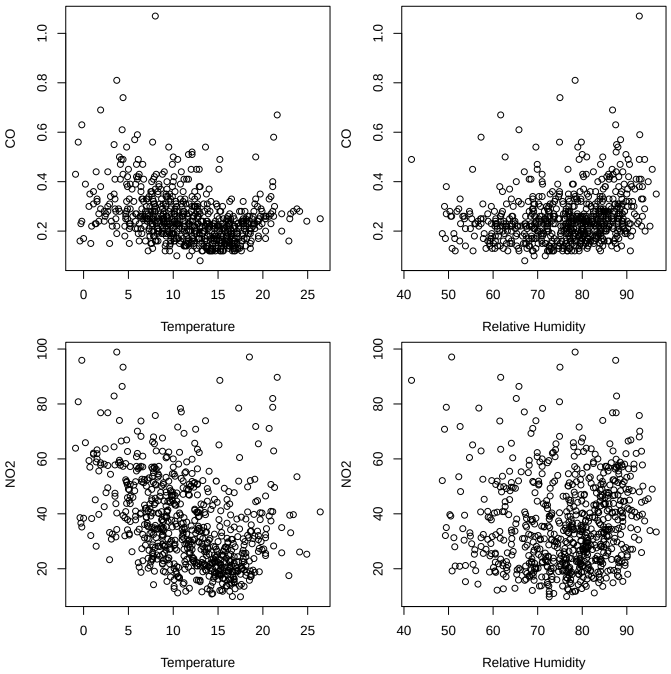

Figure A: Relationship between meteorological variables and pollutant concentrations (CO and NO_2).

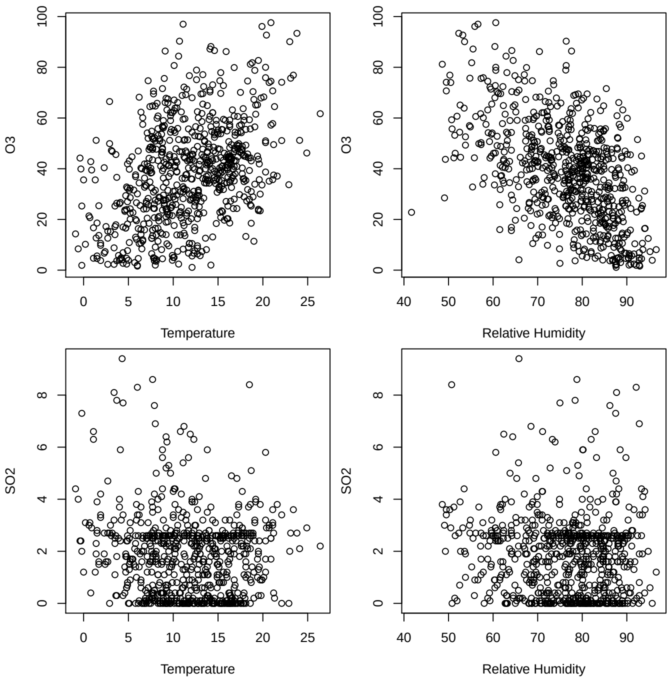

Figure B: Relationship between meteorological variables and pollutant concentrations (O_3 and SO_2).

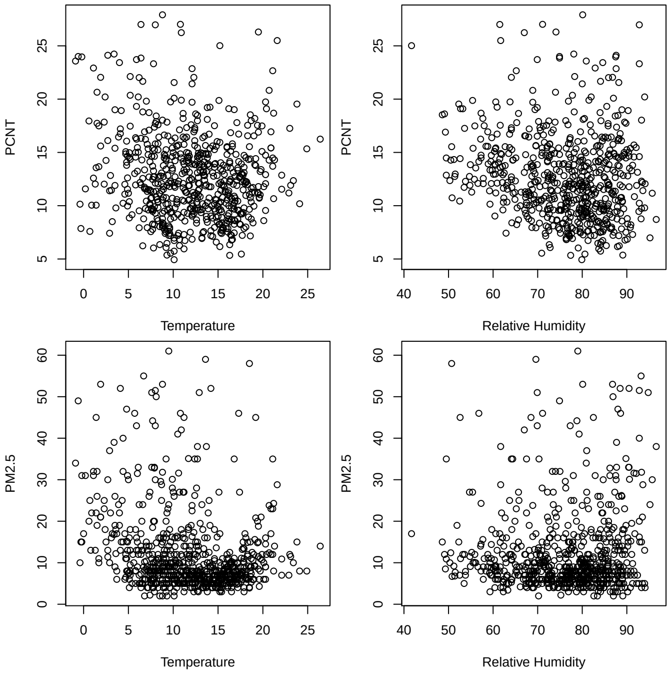

Figure C: Relationship between meteorological variables and pollutant concentrations (PCNT and $PM_{2.5}$).

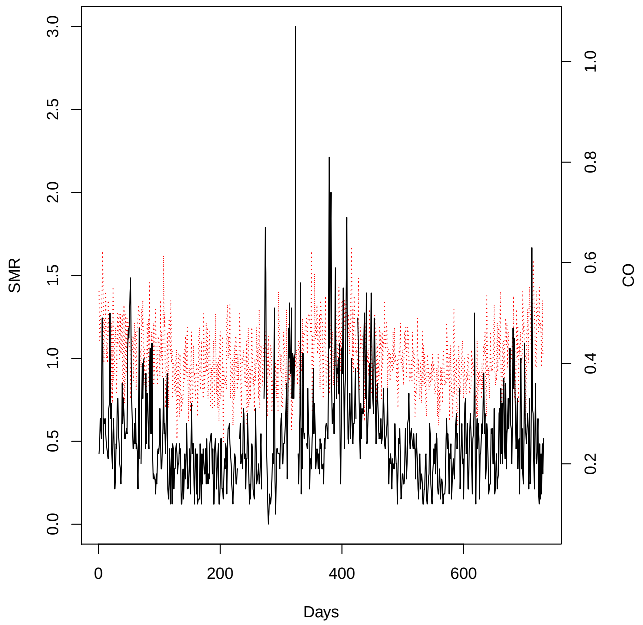

Figure D: Time series of daily mortality (red) and CO concentration (black).

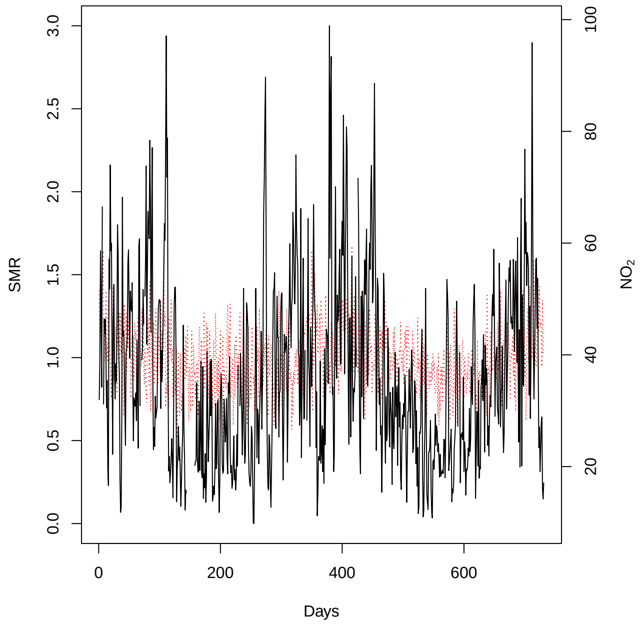

Figure E: Time series of daily mortality (red) and NO_2 concentration (black).

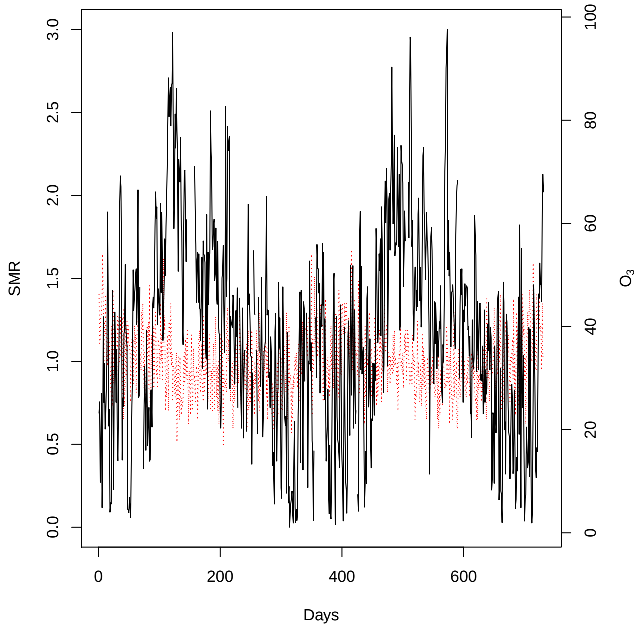

Figure F: Time series of daily mortality (red) and O_3 concentration (black).

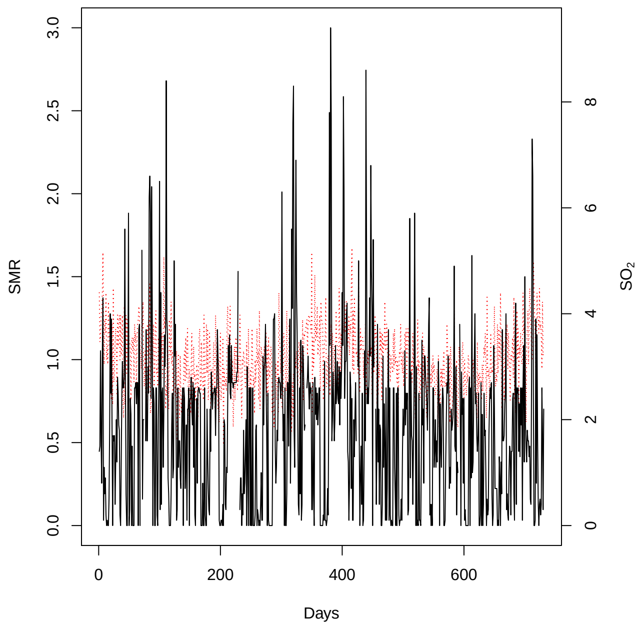

Figure G: Time series of daily mortality (red) and SO_2 concentration (black).

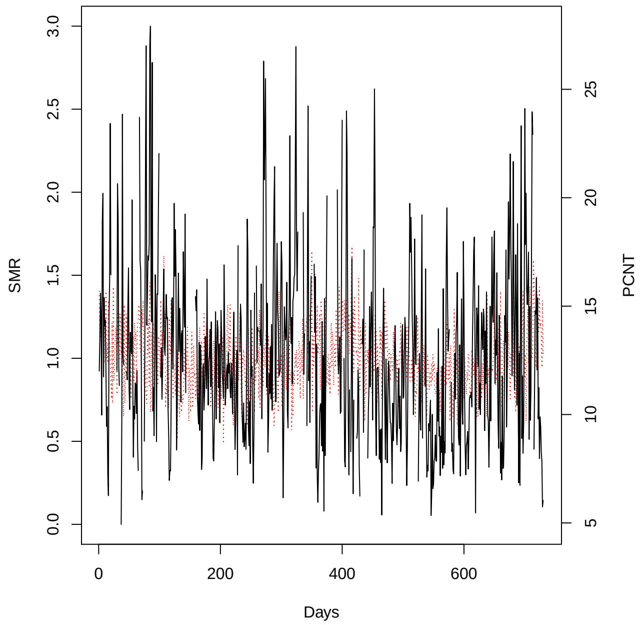

Figure H: Time series of daily mortality (red) and PCNT (black).

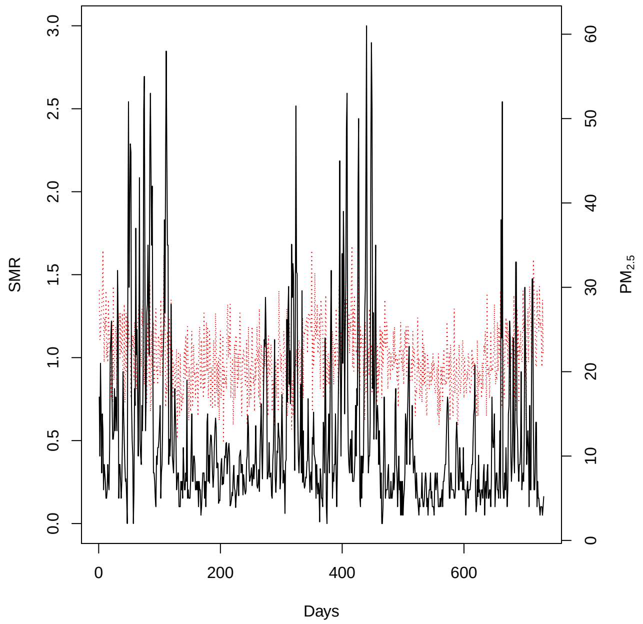

Figure I: Time series of daily mortality (red) and $PM_{2.5}$ concentration (black).

Convergence checks and model evaluation

Figures J–L present the trace, density plots and autocorrelation for the health effect parameters $\boldsymbol{\beta}$. They show good mixing between the two chains and autocorrelation close to zero for lags higher than 1, which is expected in case of convergence.

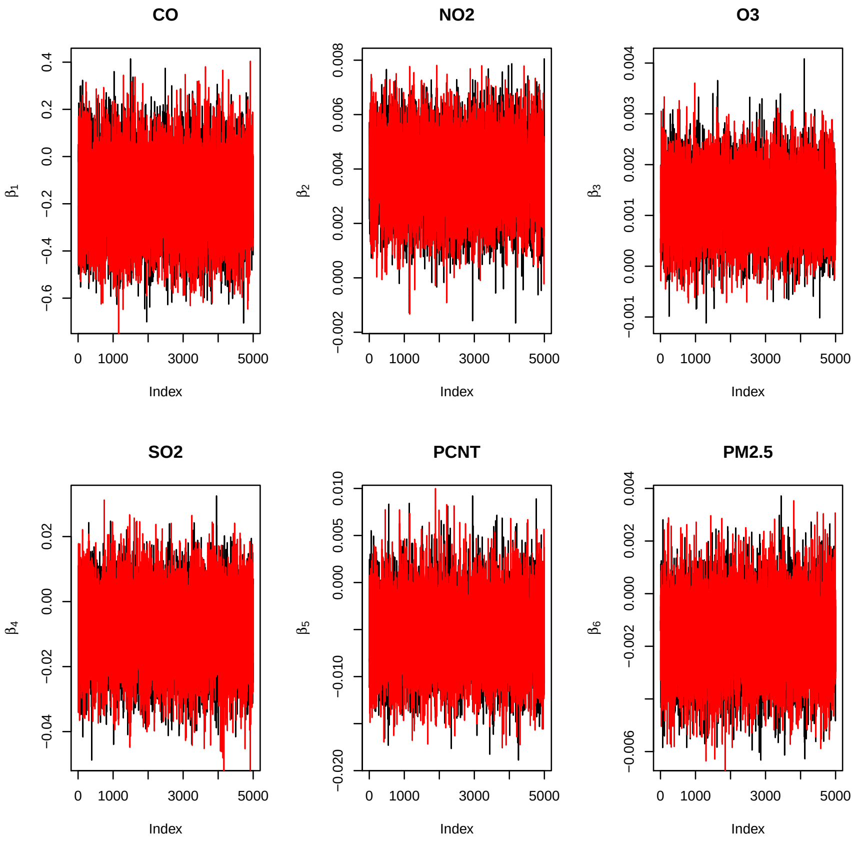

Figure J: Trace plots for the health effects of the six pollutants.

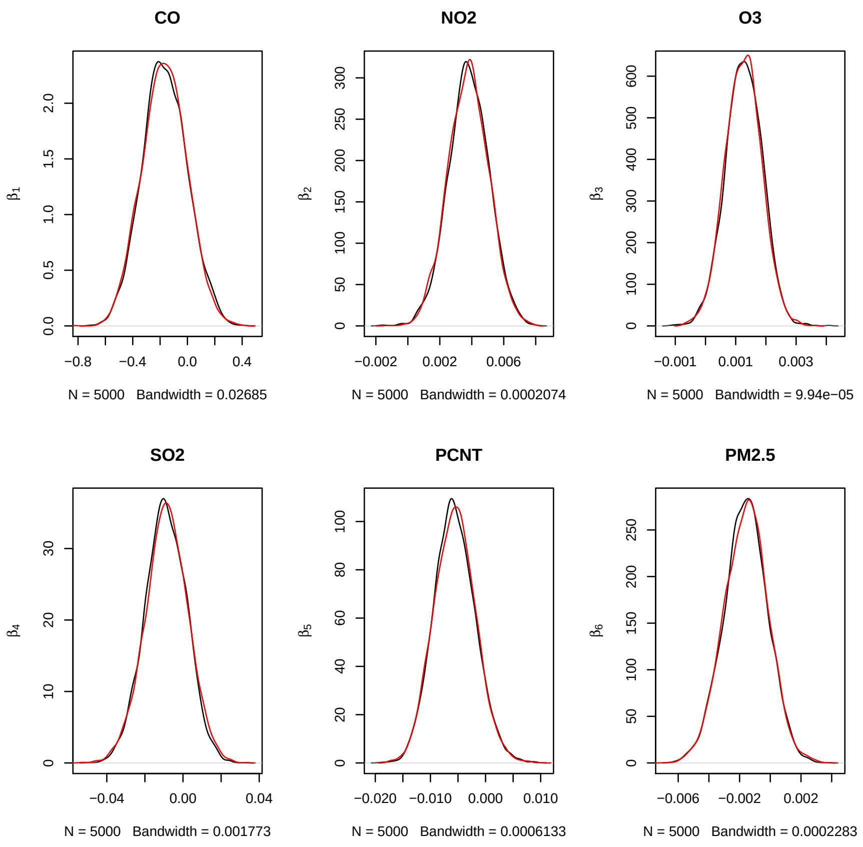

Figure K: Density plots for the health effects of the six pollutants.

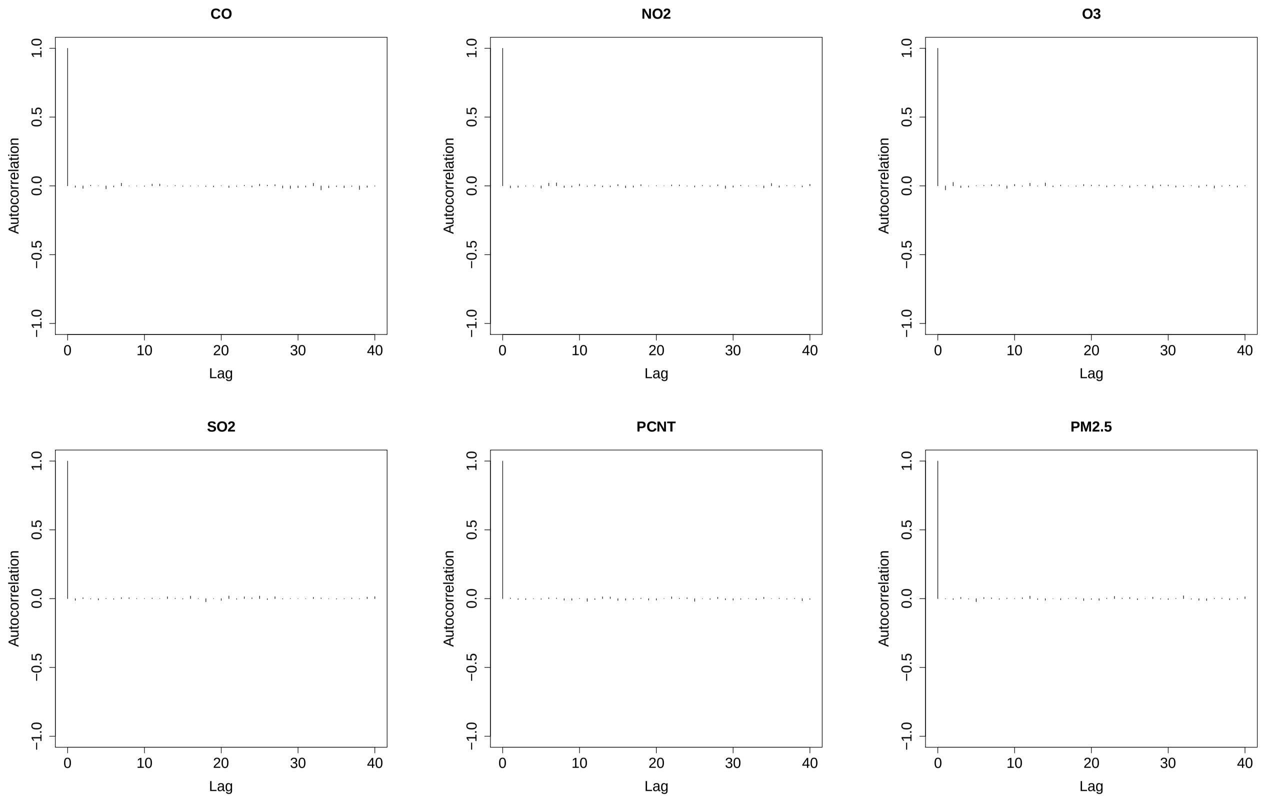

Figure L: Autocorrelation plots for the health effects of the six pollutants.

To evaluate the performance of the model in explaining the temporal trend of the pollutant concentrations, we calculated the posterior predictive values Y_{pt}^* and the residuals as:

$$\mathrm{res}_{Y_{pt}} = \int (Y_{pt}^* - Y_{pt}) p(Y_{pt}^* \mid \mu_{pt}, \sigma_p^2) p(\mu_{pt}, \sigma_p^2 \mid Y_{pt}) d(\mu_{pt}, \sigma_p^2).$$

In a similar way we estimate res_{O_t} to assess the model performance on the temporal trend of O_t. Figure M presents $\mathrm{res}_{Y_{pt}}$ and res_{O_t}, showing that they are scattered around 0 with no evidence of a residual pattern.

Figure N shows the comparison between the measured concentration Y_{pt} and the posterior mean of the estimated latent concentration μ_{pt} for $p = 1, 2, \ldots, 6$. It can be appreciated that the measured and estimated values are in good accordance and that at the same time there is a degree of shrinkage in the estimated posterior means, which is particularly visible on the SO_2 and PCNT metrics. These two metrics are also showing the highest posterior mean for the measurement error variance σ_p^2, equal to 0.66 and 0.59 respectively (see Table 4 in the main paper).

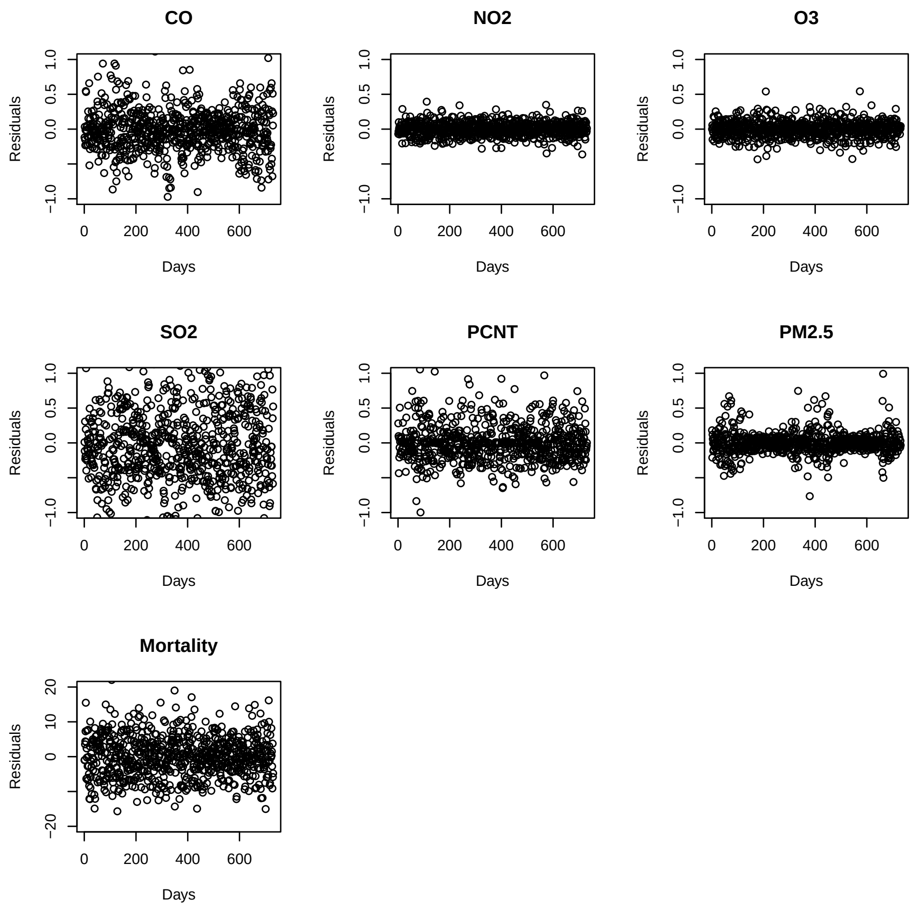

Figure M: Plots of the residuals from the pollutant ($\text{res}_{Y_{pt}}$) and health (res_{O_t}) models.

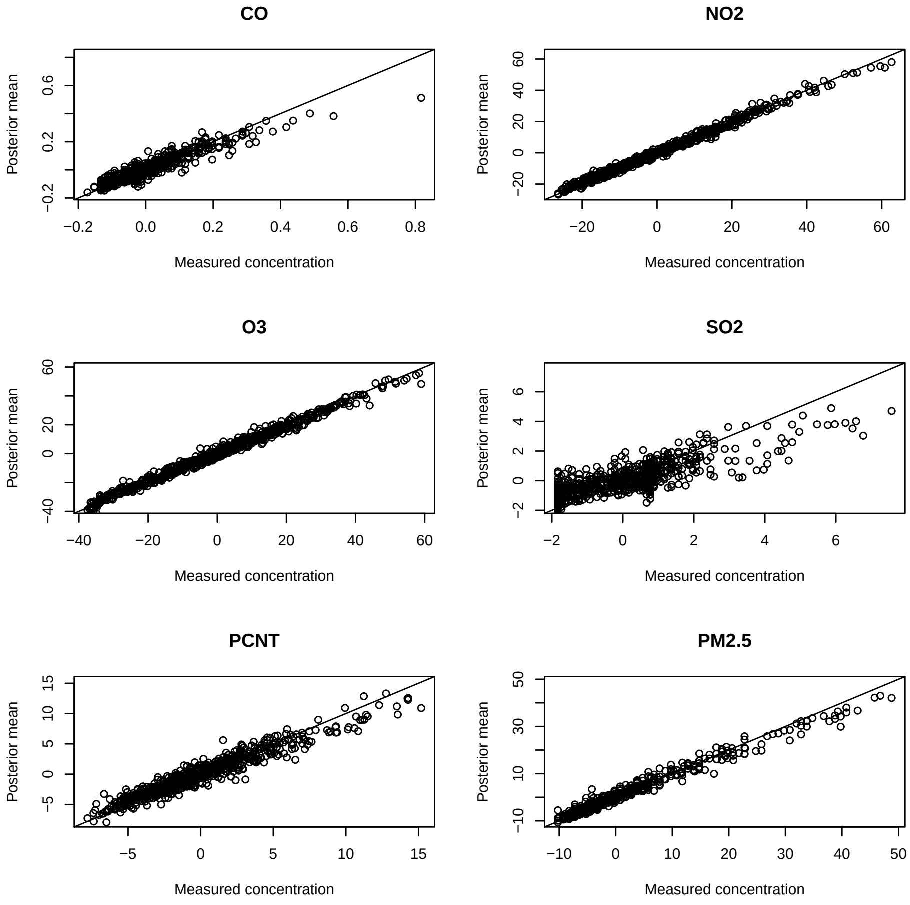

Figure N: Measured pollutant concentrations (Y_{pt}, x-axis) vs posterior mean of the estimated concentration (μ_{pt}, y-axis).

Sensitivity Analysis

In the following section we present the results of two sensitivity analyses: (i) increasing the number of knots of the splines on time, temperature and humidity (Table A), and (ii) changing the prior on the regression coefficients and on the precision parameters (Table B). Generally the results remain consistent with the modelling changes, which suggest robustness of the modelling framework.

Pollutant		IQR	Multi-pollutant model % Increase (95% CI)	
CO	(mg/m^3)	0.10	-1.49	(-4.88, 1.75)
NO_2	$(\mu g/m^3)$	23.65	10.06	(2.79, 18.53)
O_3	$(\mu g/m^3)$	26.85	2.47	(0.14, 6.04)
SO_2	$(\mu g/m^3)$	2.20	-1.41	(-6.60, 3.68)
PCNT	(p/cm^3)	5.18	-3.77	(-7.73, 0.25))
$PM_{2.5}$	$(\mu g/m^3)$	8.00	-1.80	(-4.24, 0.50)

Table A: Percent increase in mortality for an IQR change in pollutant concentrations: 9 knots on the temperature and humidity splines and 14 knots on the time spline; same prior specification as the main model; DIC=9328.

Pollutant		IQR	Multi-pollutant model % Increase (95% CI)	
CO	(mg/m^3)	0.10	-1.58	(-4.54, 1.64)
NO_2	$(\mu g/m^3)$	23.65	9.79	(3.06, 17.38)
O_3	$(\mu g/m^3)$	26.85	3.13	(0.15, 6.54)
SO_2	$(\mu g/m^3)$	2.20	-2.07	(-6.92, 2.79)
PCNT	(p/cm^3)	5.18	-2.99	(-6.87, 0.79))
$PM_{2.5}$	$(\mu g/m^3)$	8.00	-1.55	(-3.99, 0.85)

Table B: Percent increase in mortality for an IQR change in pollutant concentration: $\text{Normal}(0, 10^{-6})$ on the regression coefficients, $\text{Gamma}(0.001, 0.001)$ on the precision parameters for the basis functions, $\text{Gamma}(1, 0.001)$ for the measurement error precision; same number of knots for the spline functions as in the main model; DIC=9405.

Hikley's λ_d statistics

We used Hikley's λ_d statistics, defined as in Goldman et al., (2011) as $\frac{|\text{mean}-\text{median}|}{\text{IQR}}$ to evaluate deviation from normality. Smaller values indicate better approximation to the Normal distribution. We found that the log transformation was improving the approximation to Normality only for some pollutants, hence we decided to standardise the concentration values on the original scale.

Pollutant	Original Scale	Log-transformed
CO	0.128	0.040
NO_2	0.126	0.020
O_3	0.018	0.288
SO_2	0.014	0.665
PCNT	0.117	0.001
$PM_{2.5}$	0.403	0.087

Table C: Hikley's λ_d statistics for normality
